# ‘Somebody stuck me in a bag of sand’: Lived experiences of the altered and uncomfortable body after stroke

**DOI:** 10.1177/02692155211000740

**Published:** 2021-03-11

**Authors:** Hannah Stott, Mary Cramp, Stuart McClean, Ailie Turton

**Affiliations:** 1 University of the West of England, Bristol, UK

**Keywords:** Altered perception, body perception, embodiment, stroke, comfort

## Abstract

**Objective:**

This study explored stroke survivors’ experiences of altered body perception, whether these perceptions cause discomfort, and the need for clinical interventions to improve comfort.

**Design:**

A qualitative phenomenological study.

**Setting:**

Participants’ homes.

**Participants:**

A purposive sample of 16 stroke survivors were recruited from community support groups. Participants (median: age 59; time post stroke >2 years), were at least six-months post-stroke, experiencing motor or sensory impairments and able to communicate verbally.

**Interventions:**

Semi-structured, face-to-face interviews were analysed using an interpretive phenomenological approach and presented thematically.

**Results:**

Four themes or experiences were identified: Participants described (1) a body that did not exist; (2) a body hindered by strange sensations and distorted perceptions; (3) an uncontrollable body; and (4) a body isolated from social and clinical support. Discomfort was apparent in a physical and psychological sense and body experiences were difficult to comprehend and communicate to healthcare staff. Participants wished for interventions to improve their comfort but were doubtful that such treatments existed.

**Conclusion:**

Indications are that altered body perceptions cause multifaceted physical and psychosocial discomfort for stroke survivors. Discussions with patients about their personal perceptions and experiences of the body may facilitate better understanding and management to improve comfort after stroke.

## Introduction

Changes to sensation, movement and perception of the body are common after stroke. These neurological impairments disturb the individual’s internal representation, or schema of the body.^1^ Most patients present with multiple impairments, with about half having as many as six to ten.^2^ Yet despite the attention and clinical reasoning given to individual patients’ clinical presentations, little is known about the impact of their altered body experiences, and how they might affect thoughts about their body, or *body image.*^3^ First-person-accounts of stroke are a direct way to understand patient perceptions and changes in body image,^4,5^ and could be important to help clinicians understand the beliefs and anxieties a patient holds about their stroke affected body. With greater knowledge of their patients’ body experiences, clinicians would understand more about behaviours that might affect an individual’s progress, engagement in rehabilitation^6^ and their life after stroke. Better insight by clinicians may also improve communication to help patients make sense of and cope with changes to their body experience.^7^

For the most part, qualitative studies of body experience after stroke have focused on single labelled impairments such as sensory loss or spatial neglect.^7,8^ While informative, impairment-based studies inevitably constrain accounts to aspects that are congruent with medical descriptions. Likewise, studies of life after stroke, tend to focus on the effects of interacting with the world external to the body, rather than on the body itself.^9^ A scoping review of 28 studies searched in February 2018 and focusing on body experience, found that stroke survivors experienced the body as feeling strange, effortful, uncontrollable and hard to make sense of.^3^ Across the studies reviewed, little attention was paid to discomfort, or the negative feelings or behaviours resulting from changes to body image, which might require intervention.

In a pilot study, discussions with patients highlighted the potential for altered body perception to impact comfort. The concept of comfort has evolved within nursing to provide insight into patients’ experience in physical, psychological and social ways, rather than just in terms of bodily pain,^10^ It is viewed as a marker for ‘*acceptable standards of care*’^11^ and also a potential way to improve patient experience and outcomes.^10^ The aim of this study was to explore stroke survivors’ experiences of their bodies and determine whether their altered body caused discomfort in physical, psychological and social terms. We also sought to find out how participants coped, and whether they felt their discomfort warranted clinical intervention.

## Method

This qualitative study adopted an interpretative phenomenological approach. The approach aims to provide rich insights into how individuals makes sense of their personal lived experiences.^12^ As our focus was the participants’ experience of their stroke affected bodies, we kept in mind a philosophy of embodiment inspired by Merleau-Ponty, which suggests the body and mobility are the primary means through which we perceive and understand the world.^13^ This, together with findings from a scoping review of the literature, which highlighted the strangeness of the post stroke body experience,^3^ and the use of a lens of comfort, provided a foundation for the investigation. By applying a holistic construct of comfort^10^ encompassing physical, psychological and social comfort, we were able maintain focus on issues which were of concern to patients.

The study was informed by a patient partners group of three stroke survivors who experienced altered body perceptions. They informed the interview schedule, participant engagement and dissemination of findings. The qualitative research checklist (COREQ) was used during study development to ensure the protocol and methods were rigorous.^14^ Ethical approval was granted by the University of the West of England Research Ethics Committee (REF No: HAS/16/03/114, 21st March 2016).

Participants were recruited between March and October 2016. A sampling frame was drawn up to diversify participant characteristics in terms of age, gender, ethnicity, time since stroke onset and independence. Independence was established using the Modified Rankin Scale.^15^

Participants were eligible for the study if they had experienced cerebral ischemic or haemorrhagic stroke at least six months previously, were able to communicate verbally and were, according to self-report, experiencing any motor, sensory, or spatial attention impairments as a result of stroke. Participants experiencing conditions other than stroke, which may cause altered body perceptions, such as rheumatoid arthritis or diabetic neuropathy were excluded. Participants experiencing significant cognitive difficulties which may have affected their ability to provide informed consent were also excluded. Decisions about capacity were based on the researcher’s judgement of the individuals’ ability to retain information and whether they were able to comprehend the requirements of the study.

The researcher (HS) visited local stroke support groups where 86 stroke survivors were provided with information. Thirty-seven expressed interest in the study and were subsequently contacted by phone to determine their eligibility and to see if they would like a visit to fully explain the study. Following the visit, they were given time to think about taking part, before being contacted by phone again to ascertain their agreement. Nineteen of the 37 interested people did not meet the selection criteria; three others decided not to participate. The remaining 15 consented to participate. To increase recruitment and variation in line with the sampling frame, a charity for South Asian women was approached. This led to just one more participant being recruited. However, a sample size of 16 participants was considered sufficient to allow exploration of a range of altered body perception experiences whilst ensuring the data set could be explored in depth.

Participants gave written informed consent prior to being interviewed. Qualitative semi-structured interviews were conducted by HS, a female, early career researcher with experience in psychological interventions and stroke. In line with a phenomenological approach, interview schedules were designed to be participant focused, utilise simple, open questions and involve minimal direction from the interviewer (Supplemental File A).

To enhance participant engagement and data generation, pilot interviews were conducted. These were carried out with two patient partners and tested the flow and wording of the interview schedule. Consequently, the interview schedule was altered from a compartmentalised approach, which sought to understand each body change and determine its impact, to an interconnected approach, which allowed participants to talk more freely about the relationships between body changes and psychosocial issues. Interview questions focused on experiences of body changes and discomfort from physical and psychosocial perspectives, experiences of managing discomfort, engaging with health professionals, and the need for clinical support to address any discomfort.

Interviews with participants were conducted in participants’ homes and were audio-recorded. Twelve interviews were conducted one-to-one, three with family members present and one via an interpreter. The interpreter was experienced in working with people with stroke, knew the participant well and was briefed on how to engage in research interviews. This interview was interpreted from English to Urdu, then the English questions and responses were transcribed. The audio recording was also interpreted with an Urdu speaking researcher, to ensure concepts were accurately relayed by the interpreter to the participant, and to ensure that meaning was not lost through the process.

Field notes were used to capture the interviewers’ feelings before and after each interview, alongside reflections about the ‘positionality’ of the interviewer, which changed with each participant, depending on whether a previous relationship existed, demographic factors such as the age, gender and profession of the interviewee, how the interview flowed, participant’s environments and their demeanour.

An interpretive phenomenological analysis (IPA) was conducted and the findings were presented thematically to elucidate shared experiences. IPA seeks to represent the individual’s perspective of their experience whilst transparently acknowledging the role of the interviewer in interpreting and making meaning from their story.^16^ This allows the interviewer to interpret participants’ language, context and ‘ways of being’ to explore the underlying meaning of participant narratives of which they may not be consciously aware.^15^

First the audio data were transcribed verbatim; the first 12 interviews by HS and the remaining four by a transcription company. Qualitative studies sometimes use respondent validation, however in this study transcripts were not returned to participants for checking to avoid participants reading emotionally sensitive narratives without researcher support.

Following transcription, the data were then pseudonymised and analysed alongside the reflexive notes, assisted by the use of NVivo10 software. Firstly, interviews were re-read to familiarise the data, then the first interview was coded into broad descriptive codes to cluster the participant’s experience. Remaining interviews were read and coded in the same way, and as new areas emerged, the previous data were recoded. Salient themes were identified and recoded into more detailed sub-codes which used linguistic analysis, reflexive field notes and diagrams. Although commonality was found between cases, exploring each case in-depth, ensured the nuance and unique meaning of individuals’ experiences was not lost (Examples of coding contained in Supplemental File B).

Two coded interview transcripts were examined by a second author (SM) to ensure agreement of coding themes. During the more interpretive and reflexive phases the themes were repeatedly interrogated and refined via discussion with the whole team to ensure a robust and transparent approach (Example of reflection contained in Supplemental File B).

The homogeneity of the data meant the majority of participants’ accounts fitted into the broad areas identified. Quotes were selected and reported with care to retain focus on context and meaning. The final themes presented relate to four broad encompassing body experiences and retain focus on the physicality of the body changes and their psychosocial effects.

## Results

Participants’ details and their characteristics within the sampling frame are summarised in Table 1. All but one of the participants were younger than 75; 10 were men; only two participants were moderately to severely disabled and were unable to carry out activities independently; half of the participants were more than two years post onset of stroke; one participant was Asian Indian; the rest were White British. No participants dropped out of the study. Seven participants were known to the interviewer prior to the study due to her previous role working for the stroke charity. The duration of the interviews ranged from 33 to 106 minutes (median 73 minutes).

**Table 1.** Participants’ characteristics.

Participant pseudonym	Gender	Age	Time since onset	Affected side	Modified Rankin score*	Reported impairments and mobility
Toby	M	46	19 months	Left	1	Restricted movement/body awareness. Uses stick.
Johan	M	66	11 months	Left	1	Restricted movement/body awareness. Uses walker.
Alistair	M	72	11 months	Left	1	Restricted movement/body awareness. Uses stick.
Becky	F	58	9 years	Right	1	Restricted movement/body awareness. Uses scooter outside.
Michelle	F	49	4 years	Right	1	Restricted movement/body awareness. Uses walker outside.
Tim	M	65	8 months	Right	1	Restricted movement/body awareness/pain. Walks short distances outside independently.
Stuart	M	60	20 months	Right	1	Restricted movement/body awareness. Walks short distances outside independently, sometimes uses stick.
Daniel	M	79	11 months	Left	2	Restricted movement/body awareness. Uses walker inside, cannot walk outside.
Dave	M	65	18 months	Left	1	Restricted by pain and bodily fatigue. Can walk independently.
Amal	F	54	8 years	Left	2	Restricted movement/body awareness/pain. Uses wheelchair.
Marc	M	64	2 years	Left	1	Restricted movement/body awareness/pain. Uses stick.
Sarah	F	56	23 years	Right	1	Restricted movement/body awareness. Uses stick.
Joel	M	64	2 years	Left	1	Restricted body awareness/coordination. Walks outside independently.
Leah	F	51	21 years	Right	1	Restricted movement/body awareness/pain. Uses stick.
Tom	M	56	10 years	Left	1	Restricted movement/body awareness. Uses stick.
Lou	F	39	17 months	Right	1	Restricted body awareness. Walks outside independently.
Total	10 (Male)	Median 59	8 (<2 years)	9 (Left)	14 (Scored: 1)	15 (Restricted mobility and/or body awareness and/or pain)
	6 (Female)		8 (>2 years)	7 (Right)	2 (Scored: 2)	1 (Pain only)

* Modified Rankin score.^10^

1: No disability to slight disability. Able to look after their own affairs without assistance, may be unable to carry out all previous activities.

2: Moderate to severe disability. Requires help in some or many activities. May or may not be able to walk independently.

**Figure 1.**
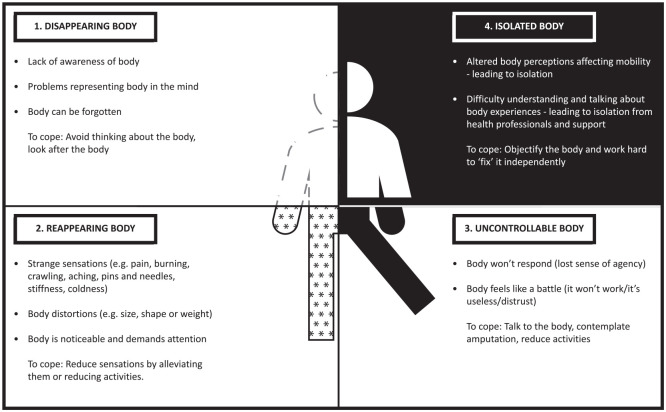
Diagram of themes and subthemes.

Four themes were identified which reflected shared body experiences: The ‘disappearing body’, the ‘reappearing body’, the ‘uncontrollable body’ and the ‘isolated body’ (Figure 1). The first three themes drew together introspective reflections on an uncomfortable body which felt as if it did not exist; felt hindered by strange perceptions and was perceived as out of control. These altered body perceptions affected individuals’ embodied sense of identity causing psychological discomfort. The ‘isolated body’ captured the social discomfort caused when participating in society whilst living with a stroke affected body. Included in the isolated body theme were the difficulty of communicating body experience with health professionals and accessing support to ameliorate uncomfortable body experiences.

Most participants described a body which they could not perceive in the same way they had done prior to the stroke, we called this *the disappearing body*. Participants used terms such as: ‘missing’, ‘not mine’, ‘forgotten’, ‘not there’, ‘amputated’, ‘doesn’t exist’, ‘subdued’, ‘half’ or ‘dead’, to describe affected body parts. For the most part, these changes to awareness and sensation were not physically uncomfortable and were easy to ignore or forget; like the parts of the body unaffected by stroke, they receded out of conscious awareness.

*“[my arm feels like] it’s just away somewhere else . . . you know unless my hand slips down . . . like that, between my leg, I forget that it’s there” (Marc, 2 years post-stroke)*

Frequently, the experience of perceptual absence only became noticeable when the body was brought into conscious awareness. This occurred when the body was injured or limbs needed protecting, moments when others interacted with the body and when the body was needed for action.

*“they [nurses] were like putting pins in me and I couldn’t really feel the pins in me down my arm. . .it was a bit weird. [I felt] a bit scared . . . [it’s like] ‘oh, okay, what’s happening?’” [Laughter] (Lou, 39, 17 months post-stroke)*

Moments like these forced the individual to notice and confront their changes to body awareness. This changed relationship with the body was emotionally uncomfortable at times.

*“[It feels] like somebody stuck me in a bag of sand, I don’t know why. . . half your body is sticking out. . .the other side’s sticking in, the affected side. . . it can make me feel I want to cry sometimes. . . other times you don’t think about it” (Tom, 56, 10 years post-stroke)*

The incomprehensible nature of the perceptual loss led many to express the physical sensations in terms more usually linked to cognitive ideas of identity – *‘it’s like it don’t belong to you. . . . I’m a person of two halves, like a split personality’ (Becky, 58, 9 years post-stroke).*

Frequently participants used third person or negative language towards body areas. We interpreted this as an unconscious mechanism of objectification used to create separation between the individual’s sense of identity and their loss of body perception.

*“I can obviously move all my limbs, but I can’t really feel them, it’s like . . .having Novocaine. . . it doesn’t feel like me anymore [laughs]. . . . it’s like [exhale/sigh] a parasite [laughs]. . .it is part of me but it’s not what it was. . .[pause] it’s who I am now. . .I don’t hate it, it’s just that that’s what it is. . . I don’t have to like it. . . [laughs]” (Leah, 51, 20 years post stroke)*

Other coping strategies were also deduced: participants tended to laugh off, dismiss and avoid the bizarre nature of their altered perceptions. These were interpreted as a means to evade the emotional discomfort of describing experiences which felt weird, embarrassing, hard to make sense of and difficult to communicate.

*[Imagining his body with his eyes closed] “It would be half a body. I would be cut in half [down the middle] or whatever. . . [on the left there would be] nothing. . . it would be empty. . . [Interviewer: How does that make you feel?] Worse than I do if I don’t think about it [laughs]” (Marc, 64, 2 years post-stroke)*

At the same time as experiencing perceptual absence, it was common for participants to describe altered perceptions in which the body ‘reappeared’. *The reappearing body* became impossible to ignore and demanded conscious attention. Experiences of perceived differences in body size, shape, weight, instability, pain and strange sensations such as pins and needles, stiffness and coldness were common and affected daily activities.

*“the arm can feel very cold sometimes, I noticed between the two there’s a difference in temperature. . . the fingers. . . its constantly tingling. . . my little toe on the right side all the way. . . up to my ankle. . . can get hypersensitive and also, I feel as though my ankle’s broken. . . it just feels painful. . . I know it isn’t broken. . . and it’s not swollen, but it just gives me the wrong information” (Tim, 65, 8 months post-stroke)*

These sensations were noticeably physically uncomfortable which drew attention to the body. This led to attempts to make sense of why the body felt strange and to alleviate uncomfortable sensations, for example by restricting movement, distracting from pain, trying to keep warm or taking pain medication.

*“I try to avoid putting it places where it’s likely to be hurt. . . if I’m mowing the lawn, then I would use my right arm to lead when the mower is going away from me, rather than have this mower wrench my left arm away from me. . . I’ve learnt to accommodate it. . . I know how to avoid the pain” (Alistair, 72, 11 months post-stroke)*

Most participants described effortful experiences of bodily heaviness, which could build up during the day or occur out of the blue. Limb weight felt equivalent to having a *‘young child grabbing hold of your knee’* (Alistair, 72, 11 months post-stroke) or like *‘dragging a 10lb dumbbell behind you’* (Tom, 56, 10 years post-stroke) and required so much ‘*perseverance. . . to make it go. . .It probably does use up more calories’* (Sarah, 56, 23 years post-stroke). For a few this led to the body feeling stuck or collapsing, which inevitably led to negative feelings and affected social opportunities.

*“I suppose I do collapse. . .when I’m walking. . . I am saying ‘I will walk on’, but my legs are saying ‘if you want to walk on, carry on without us’ [laughs]. . . I am not going to give up, but it makes you feel like giving it up sometimes” (Daniel, 79, 11 months post-stroke)*

Frequently there were no words to convey the abnormal and uncomfortable physical bodily experience and participants had to rely on creative metaphors or simply stated ‘it’s weird’ to dismiss the strangeness of their experiences. Leah expressed that her altered perceptions made her body feel like the *‘special effects suit of the Creature from the Black Lagoon.’*

*“That side. . . feels bigger. . .monster-ish [laughs]. . . [pause] it’s like a marshmallowey, come sort of like bloated. . . not human. . . [laughs]” (Leah, 51, 21 years post-stroke)*

The use of metaphors was interpreted as a useful way to convey the strangeness of their perceptions and to objectify their body experience. At times this objectification was physically validated because the sensations being felt in the body were incongruent with what was being visually observed. This conflict forced individuals to focus their attention on the body perceptions that were hard to make sense of and difficult to manage.

*“[Its] like you’re falling over on your ankle. . . my brain’s saying ‘Your foots not flat’. . . although I don’t feel it. . . [it feels] strange, it doesn’t make sense. . . [It’s uncomfortable] ‘cause it feels like you’re going to fall over. . . but you’re not. . . If you’ve ever gone in the sea and it’s gone a bit boggy underneath. . . and your feet. . . like jelly and moving. . . I guess it’s a bit like that really. Your eyes tell you one thing and your brain’s shouting something else. . .” (Becky, 58, 9 years post-stroke)*

*The uncontrollable body* encompassed experiences from both the disappearing and the reappearing body, such as strange perceptions or perceptual loss, alongside a lack of bodily response. This led to all participants describing difficulty moving, using or controlling the body, which affected their independence in activities, feelings about the self and relationships. Participants described an inability to match up their intention to move the body with achieving an appropriate response. This conflict catapulted the uncooperative body into their attention.

*“I feel like I ought to be able to do everything I could do. . .and just suddenly [the feeling] it’s not there on the right side. . . it’s that gap, that’s the thing. . . you can’t find. . .anywhere in your brain, the signal that will move the arm. . . it feels like it should be able to do it, but somehow it just can’t. . .” (Stuart, 60, 20 months post-stroke)*

Participants would attempt to override the rebellious body by instructing it to engage in movement through a conscious dialogue. We interpreted this as being a way to compensate for mismatched perception.

*“I’m having a conversation with the person I call Jiminy Cricket. . .he’s on my left shoulder. . . saying ‘move your left leg’ and stuff. . .he’s an imaginary person obviously. . .he never answers back. . . he just says ‘do it’ and you do it [laughs]. . . well that feels worse than the shoulder feels. . . because you’ve got no control over him. . .” (Marc, 64, 2 years post-stroke)*

Engaging in this dialogue required an uncomfortable feeling of ‘*disabling effort*’ (Sarah, 56, 23 years post-stroke) to achieve body function. It was construed that emotionally the body had become a battle. The sense of frustration with the unresponsive body merged with feelings of fear and distrust that the body could not meet the expectations and intentions of the mind.

*“I no longer have a correct sense of what’s vertical. . . I need to be slightly off-balance in order to be vertical. . . and I’m therefore afraid I’m going to fall over. . . that leads to fear and anxiety. . . so that slows me down. . . walking across a carpark or something is terrifying. . . I’m in free space. . . and I think if my balance was right, I could make the rest work and I don’t know how on earth to get my brain to get its sense of balance right again” (Johan, 66, 11 months post-stroke)*

To cope, participants learnt to override the untrustworthy body input and use cognitive strategies to risk assess or reduce activity within their environments and reduce injuries from falls. However, despite these plans and years of coping with these experiences, participants continued to live with fear and a sense of feeling unsafe.

Most participants maintained a detached perspective towards their loss of bodily control and tried to fix it by working hard in rehabilitation, which, it was interpreted, helped them avoid feelings of uselessness and retain a feeling of hope. However, three male participants (Toby, Johan and Marc) illustrated the extent of their sense of detachment towards the uncontrollable body by considering, at times, amputating the affected limb; consequently eliminating the reminder of the body part which created emotional discomfort.

*“I’m not in discomfort if you see what I mean. . . I’m only in discomfort when I can’t move it. . . it’s all a waste of time this arm now. . . because it won’t work, it’s there but it won’t work [laughs]. . . I often think ‘well should I just cut it off one day’. . . get it out the way. . . but then I think, well my shoulders still there. . . [it makes me feel] bad” (Marc, 64, 2 years post-stroke)*

*The isolated body* encompassed experiences of reduced mobility caused by altered body perceptions, such as changes to feeling balanced, distorted perceptions of the shape, heaviness or stiffness of limbs, the experience of pain and altered sensations and an inability to get the mind to move the body. Participants felt they were isolated by their immobility, as these body perceptions effected their ability to engage in society which caused psychosocial discomfort.

*“having a leaden telegraph pole instead of a leg is very depressing. . . just going out to dinner. . .I’m thinking: ‘by 10 o’clock I’ll hardly be able to walk’. . . it’s a bit like Cinderella’s coach turning back into a pumpkin” (Johan, 66, 11 months post-stroke)*

Participants commonly reported that immobility caused by altered body perceptions was their biggest problem and said this would be their main focus for treatment, if they could have it. However, participants were uncertain if any treatments existed, and often laughed at this idea.

*“Nobody has explained to me, the physiology of ‘Why does it feel as if it needs oiling? What is it?’. . . So, I look for signs of improvement. . . What I’d like is just to be able to get up in the morning and have no heavy leg or stiff arm. . . just to get on with it. That’s why I kind of work away at the gym thinking there may come a break through moment when I get up one morning. . . and this leg doesn’t feel heavy and I can. . . walk smoothly. . . the lack of mobility in the left side, that’s. . . half of the problem. . .” (Alistair, 72, 11 months post-stroke)*

Uncertainty about the causes of immobility and their prospects for recovery was exacerbated as participants found stroke specialist support inaccessible. For many, these negative attitudes developed following unsatisfactory encounters predominantly with GPs and hospital consultants, but also with nurses and rehabilitation staff, in which participants felt that health professionals did not have the time, specialist stroke knowledge or resources to help them.

*“Health professional? What? Who? There isn’t anyone [laughs]. . .my GP says: ‘It looks like the [hospital] have dumped you onto the GPs. . . but we haven’t got the time or the resources to help you’. . . There’s nothing at all. . . isolated. . . you’re left to your own means. . . so this National Health, so far has been absolutely useless for me.” (Tim, 65, 8 months post-stroke)*

Others felt that the treatments to alleviate their altered body perceptions did not exist.

*“I suppose I do collapse, like the undercarriage has given way. . .I had a lot of visits now, from the physio and they concluded that they can’t do anything for me. . . because they don’t know what’s wrong. . . I’ve done all the exercises they told me to do, but no. . . I am getting on a bit, but if there is some sort of cure, or some sort of way of reducing the discomfort I’ve got [but] that’s all there is” (Daniel, 79, 11 months post-stroke)*

Participants found communicating their body experiences to clinicians problematic due to difficulties in finding the words to describe their experiences, and uncertainty about how health professionals might respond to the perceived strangeness of body sensations. *‘I’ll be diagnosed as being schizophrenic or something. . . or depressed. . . they’d think I’d had a screw loose. . .’* (Marc, 64, 2 years post-stroke). This lack of clinical input left participants with ongoing uncertainty about the reasons they experienced altered body perceptions, how best to engage in rehabilitation, or how to alleviate sensations which might reduce their discomfort. Participants tended to personalise the lack of rehabilitation available to them and developed feelings that they were a burden on the health services, or not worth rehabilitating. Their perceived abandonment was interpreted as an external validation of negative, troubling feelings towards the post-stroke body and self.

*“They [NHS clinicians] say ‘Yeah’ but they don’t listen. . . they’re just trying to shut you up. . . they don’t really care. . . like a cattle market. . . you’re just the next one in the ring. . .if that makes sense. . . just get used to it don’t you?. . . They just say: ‘It was the strokes’. . . They wrote you off almost.” (Becky, 58, 9 years post-stroke)*

In the face of these experiences, participants, particularly those who were earlier post-stroke, were hopeful that if they worked hard independently, they may improve their problematic body perceptions. It was interpreted that the idea of ‘fixing’ the body indicated participants’ sense of detachment between body and self. A few participants, many years post-stroke, suggested they had accepted their altered body. However, interpretation suggested that this idea of reconciliation between body and mind was complex; rather than feeling united, they had instead prioritised their sense of identity over their problematic body to alleviate any emotional discomfort caused by their altered body perceptions.

## Discussion

This qualitative study explored stroke survivors’ experiences of their bodies to determine whether their altered body perceptions caused discomfort in physical, psychological and social terms. The findings showed that individuals could experience many, sometimes conflicting, altered perceptions at the same time. Stroke-affected body parts could be unnoticed, as if they were absent, especially when the person was still. This lack of sensation or registration of body parts was not physically uncomfortable but could be psychologically troubling to think about. Participants also experienced physical and psychological discomfort due to strange sensations or body distortions which demanded their conscious attention. The body was experienced as frustrating when it failed to respond easily to the intention to move. Participants were continually reminded of their uncontrollable body and felt socially isolated by it. Participants also felt isolated from clinical support due to their uncertainty both in describing their body experience. This, and their belief that clinicians had no treatments or expertise to help them, resigned them to living with discomfort.

Our findings resonate with descriptions of altered body perception in studies with more narrowly defined criteria, such as the experience of sensory loss in the arm, or at specific time points post-stroke.^8,17^ These studies have highlighted the feelings of strangeness of the body, the effort involved in using limbs and the body being experienced as uncontrollable. These studies, like ours, have observed stroke survivors’ psychological reactions to the stroke affected body, of disowning it, separating themselves from it and finding it, hard to make sense of.^4,18,19^ The coping strategies reported in our phenomenological study have sometimes been explicitly expressed, for example, working hard at the gym to fix the body; or talking to the limbs to get the body to move. Other strategies, which may not be consciously employed, were interpreted from the data, for example talking about the body in third-person language. We deduced these reported behaviours as mechanisms to create psychological distance between the person’s identity and their body. This kind of objectification of the disabled body is well established in the stroke and chronic illness literature,^20^ as a means to cope when confronted with difficult ideas about the body and self.

This study highlighted the struggles of stroke survivors in expressing altered body perceptions. Our findings and the wider literature^3^ suggest that patients want more opportunity to discuss their body experience with clinicians. Clinical interactions have the potential to redirect patients’ problematic coping strategies and change attitudes towards their body image. For example, changing attitudes to the body can alter how pain is understood and perceived.^21^

Guidance for rehabilitation therapists, to help patients to adjust with resilience to their body image and identity after stroke is scarce, however there is recognition that clinical reasoning should include patients in a participatory process to make sense of their body experiences.^22^ The current lack of acknowledgement of altered body perceptions suffered after stroke is reminiscent of the clinical experience of amputees up until the end of the 20th century. Phantom limb pain and sensations were written about, but were not acknowledged as common within medical practice.^23^ Since participants in our study expressed a difficulty finding the words to describe their experiences, a good starting point for clinical practice and for further research would be to provide opportunities and tools to help patients and clinicians to have these conversations.

Most of the participants in this study were of working age and therefore younger than average for people who have had a stroke in the UK.^24^ They had all been discharged from stroke rehabilitation services. In agreement with reports about the unmet needs for people discharged from stroke care, participants articulated their struggles to access further rehabilitation and expressed a feeling of abandonment by their health services.^25,26^ Some were trying to improve their bodies through their own efforts by continuing to work on their strength and mobility; others had accepted that they had to live without expecting any change in their bodies. Nevertheless, most participants’ priority for treatment was to improve altered perceptions which they reasoned were impacting on their mobility.

Collating subjective patient information of body experience after stroke deepens our understanding of the interactions between multimodal physical and psychological influences on body image,^27–29^ which could provide useful information to guide rehabilitation. It could be that current evidence-based rehabilitation interventions might have an effect on body experience. For example, use of illusory rehabilitation techniques that influence sensorimotor perception, such as mirror therapy or virtual reality might provide a means to change altered body perceptions.^30,31^ Alternatively, psychological therapies may help with acceptance of distressing body experiences.^32^ However, it is possible that new interventions are needed to improve the comfort of stroke affected bodies. Future research for interventions to help people living with stroke should take body experience into account.

The strengths of the study were its success in capturing experiences which are difficult to describe. The involvement of patient partners helped in making sure the participants understood what we wanted to explore with them. The holistic construct of comfort facilitated a broad insight into the experience of the stroke affected body. Though this idea of comfort was understandable for participants, it was interpreted in different ways. In many instances participants did not explicitly refer to their body experience in terms of ‘discomfort’, but used descriptors related to unpleasant sensations such as pain, distortions of body size, weight or shape, frustration, anger or isolation. These experiences were interpreted and grouped by the author as relating to physical or psychosocial discomfort and were therefore indirectly construed as uncomfortable.

In answering questions about whether treatment was wanted for alleviating troublesome body experiences, it was sometimes unclear whether participants could separate rehabilitation for improving function from rehabilitation for improving comfort. This was consistently questioned in the interviews to clarify what was meant. However, there was some lack of distinction which may be because participants envisaged that improved function would, in turn, lead to a reduction in uncomfortable body experiences.

The sample size was appropriate for a qualitative study and a range of experiences were captured. However, the findings should be considered in light of the sample, who were people who attended stroke support groups, and so may have experienced opportunities to articulate stroke related body changes. In terms of diversity of stroke survivors, we did not achieve the variation we intended. Our sampling frame showed an imbalance across characteristics, with few older people, few with severe stroke and only one participant from a minority ethnic background. Further studies designed to hear the experiences of these groups and also from stroke survivors with aphasia are needed.

## Conclusion

For many stroke is a life-long condition, which leaves individuals with ongoing altered body experiences and discomfort, isolated from services and support. In this study stroke survivors articulated their struggles to access clinical interventions to reduce their discomfort. The unique and bizarre nature of many of their body experiences compounded this problem, leaving individuals uncertain of the causes of their body perceptions, how to talk about them with clinicians, and how best to manage them independently. Further research is required to explore with stroke survivors how to make sense of their altered body experience and improve their comfort. This might deepen clinical understanding of the multimodal components of body image and direct new avenues to rehabilitate discomfort after stroke.

**Clinical messages**

- Stroke survivors experience their bodies as physically, psychologically and socially uncomfortable.
- Stroke survivors find body experiences difficult to describe and want ways to communicate how their body feels to healthcare staff.
- Stroke survivors identify lack of understanding of their body experiences and want support to improve comfort.

## Supplemental Material

sj-pdf-1-cre-10.1177_02692155211000740 – Supplemental material for ‘Somebody stuck me in a bag of sand’: Lived experiences of the altered and uncomfortable body after stroke Click here for additional data file. Supplemental material, sj-pdf-1-cre-10.1177_02692155211000740 for ‘Somebody stuck me in a bag of sand’: Lived experiences of the altered and uncomfortable body after stroke by Hannah Stott, Mary Cramp, Stuart McClean and Ailie Turton in Clinical Rehabilitation

sj-pdf-2-cre-10.1177_02692155211000740 – Supplemental material for ‘Somebody stuck me in a bag of sand’: Lived experiences of the altered and uncomfortable body after stroke Click here for additional data file. Supplemental material, sj-pdf-2-cre-10.1177_02692155211000740 for ‘Somebody stuck me in a bag of sand’: Lived experiences of the altered and uncomfortable body after stroke by Hannah Stott, Mary Cramp, Stuart McClean and Ailie Turton in Clinical Rehabilitation
