# Supplementary material for: ‘Somebody stuck me in a bag of sand’: Lived experiences of the altered and uncomfortable body after stroke
Source: Clin Rehabil. 2021 Mar 11;35(9):1348–59. doi: 10.1177/02692155211000740 (PMC8358539; doi:10.1177/02692155211000740)
Supplement: sj-pdf-1-cre-10.1177_02692155211000740 – Supplemental material for ‘Somebody stuck me in a bag of sand’: Lived experiences of the altered and uncomfortable body after stroke [file sj-pdf-1-cre-10.1177_02692155211000740.pdf]

Supplementary file A: Interview guide

- How long ago was your stroke?
- How has your stroke affected you?
- What changes have you noticed in your body?
    - How does that area look in your mind's eye?
    - When do/did you notice this change?
    - What triggers it, how does it change over time?
- How comfortable are these body changes?
    - Physical impact: How does this body change affect your day to day life?
    - Emotional impact: How does this body change make you feel?
    - Social impact: How does this body change affect where you go and your relationships?
- How do you manage this body change?
- Have you told a health professional/had an intervention?
- How much impact are these changes having on your life?
- If treatment existed what would you treat?
