# Supplementary material for: ‘Somebody stuck me in a bag of sand’: Lived experiences of the altered and uncomfortable body after stroke
Source: Clin Rehabil. 2021 Mar 11;35(9):1348–59. doi: 10.1177/02692155211000740 (PMC8358539; doi:10.1177/02692155211000740)
Supplement: sj-pdf-2-cre-10.1177_02692155211000740 – Supplemental material for ‘Somebody stuck me in a bag of sand’: Lived experiences of the altered and uncomfortable body after stroke [file sj-pdf-2-cre-10.1177_02692155211000740.pdf]

Supplementary file B: Examples of coding and reflexivity

A. Three examples of coding and interpretation

1. Leah (51, 20 years post stroke)

> *"I can obviously move all my limbs, but I can't really feel them, it's like ...having Novocaine… it doesn't feel like me anymore [laughs]…. it's like [exhale/sigh] a parasite [laughs]…it is part of me but it's not what it was...[pause] it's who I am now…I don't hate it, it's just that that's what it is... I don't have to like it… [laughs]"*

Coding area	*Example comments*
Finding the words to communicate (Similes)	• *"it's like ...having Novocaine"*<br>• *"it's like [exhale/sigh] a parasite"*
Embarrassed communicating the strangeness of the body (Laughter to detract from content)	• *"it doesn't feel like me anymore [laughs]"*<br>• *"I don't have to like it… [laughs]"*
Separation between body and self (Third person language towards body, first person language towards self)	• *"it doesn't feel like me anymore"*<br>• *"it is part of me but it's not what it was...[pause] it's who I am now"*
Acceptance of body changes is complex (Negative language/tone towards body)	• *"it's like [exhale/sigh] a parasite"*<br>• *"I don't hate it, it's just that that's what it is... I don't have to like it… [laughs]"*

2. Becky (58, 9 years post-stroke)

> *"It's like it doesn't belong to you...I know it is mine, I know it's there, I know it's part of me... 'cause I can see it... it's weird but it don't exist...just like you got a zip down [draws line with her finger down the middle of her body] ... I'd say I'm a person of two halves... like a split personality."*

Coding area	*Example comments*
Finding the words to communicate (Similes, expressing strangeness)	• *"It's like it doesn't belong to you"*<br>• *"like a split personality"*<br>• *"it's weird"*

Separation between body and self (Third person language towards body, first person language towards self. Body as 'other' affects understanding of self)	• *"It's like it doesn't belong to you..."*<br>• *"I know it is mine, I know it's there, I know it's part of me... 'cause I can see it... it's weird but it don't exist"*<br>• *"I'd say I'm a person of two halves… like a split personality"*

3. Alistair (72, 11 months post-stroke)

> *"Yesterday I was feeling quite weak, heavy legged, sometimes it feels as if they're lead-like. Have you ever had a young relative grab you round the knee and hang...on and you drag your leg along?... that's kind of what it can feel like, it's really hard work y'know... and there's nobody hanging on...The leg is heavy for some reason, I don't know why... and I think well the leg is strong and I've been fortunate that I've had quite a bit of strength in the leg right from the start and it's improved... so if it's that strong why can't I just walk normally? And I don't know the answer to that."*

Coding area	*Example comments*
Finding the words to communicate (Similes)	• *"as if they're lead-like"*<br>• *"[like] a young relative grab you round the knee and hang...on and you drag your leg"*
Separation between body and self<br>(Third person language towards body, first person language towards self)	• *"The leg is heavy for some reason, I don't know why... and I think well the leg is strong and I've been fortunate"*
Making sense of altered body perceptions (Expressing uncertainty)	• *"The leg is heavy for some reason, I don't know why"*<br>• *"why can't I just…?"*<br>• *"I don't know the answer"*

B. Reflection on researcher 'positionality' and decisions around interpretation

Participants described a sense of having to take care of the separate and objectified body. The body which was lost, uncontrollable and needed consciously looking after was consistently described. Many participants responded to this bodily demand with a calm and detached response, they made light of their disobedient bodies, they cared for their sore and problematic limbs and tried not to let these changes affect their sense of self.

> *"[it is uncomfortable] when I turn over in bed and I forget to move my arm first. [I feel] a pain [laughs]... I have to turn back and pull this arm over. [It's gone] Behind my back." (Sarah, 56, 23 years post-stroke)*

> *"I try to avoid putting it places where it's likely to be hurt. So, if I'm mowing the lawn, then I would use my right arm to lead when the mower is going away from me, rather than have this mower wrench my left arm away from me... So, I've learnt to accommodate it in ways like that...so I know how to avoid the pain" (Alistair,72, 11 months post-stroke)*

I reflected that, as a mother of a six-year-old boy, participants' descriptions resonated, as they reminded me of a parent caring for a child. The objectification of their body parts made those limbs seem almost like they were another person, who they felt a responsibility to care for. At times those limbs were annoying, frustrating and hard work, but despite this, like a parent to a child, participants maintained an attitude of conscious care and forgiveness.

However, despite this parallel, I also noted how my experience influenced the lens through which I was viewing the data. As a result, I did not compile a theme around the idea of infantilising the body. Instead, I developed a theme exploring the separation between body and self and considered several subthemes which detailed the different ways of coping with a rebellious body, one of which was consciously caring for the body.
